# Progression and Resolution of a Post-traumatic Pleurocutaneous Fistula

**DOI:** 10.7759/cureus.44944

**Published:** 2023-09-09

**Authors:** Benjamin O'Brien, Aidan Farrell, Patrick Janeczko, Pranav Shah

**Affiliations:** 1 Radiology, Hackensack Meridian School of Medicine, Nutley, USA; 2 Vascular and Interventional Radiology, Jersey Shore University Medical Center, Neptune, USA

**Keywords:** subcutaneous emphysema, trauma surgery, pneumothorax, traumatic injury, pleurocutaneous fistula

## Abstract

Pleurocutaneous fistula (PCF) is a pathological communication between the pleural space and subcutaneous tissue. This rare condition occurs as a complication of infection, malignancy, and therapeutic procedures such as tube thoracostomies. PCF is typically confirmed with computed tomography (CT) imaging. There is no current literature describing the post-traumatic causes of PCF. We describe a PCF related to multiple rib fractures and its rapid improvement following the placement of a chest tube. This case emphasizes the importance of prompt CT imaging in trauma patients and radiographically illustrates the progression and resolution of a post-traumatic PCF.

## Introduction

Thoracic trauma may result in a wide range of injuries including tension pneumothorax, cardiac tamponade, hemothorax, flail chest, traumatic aortic injury, myocardial contusion, diaphragmatic tear, and pulmonary contusion [1]. In the event of high-speed blunt trauma to the chest wall, a patient may develop one or a combination of these potentially life-threatening injuries. Pleurocutaneous fistula (PCF) is an aberrant communication between the pleural space and subcutaneous tissue due to poor healing of a break in the parietal pleura [2]. Pyogenic, often mycobacterial, pulmonary infection, especially in immunocompromised patients, is a well-documented etiology of PCF [3,4]. PCF and bronchopleural fistula can both develop iatrogenically as a rare complication of tube thoracostomy placement or video-assisted thoracoscopic surgery (VATS) [2-6]. PCF has also been described in cases of malignancy, including pleural squamous cell carcinoma and following radiation therapy of breast cancer [2,7]. The clinical presentation of PCF is typically a non-specific palpable subcutaneous thoracic mass [8]. An audible high-pitched squeak during a sustained Valsalva maneuver may be heard as an indicator of an air leak [9]. Diagnosis of PCF is made radiographically, usually via computed tomography (CT) imaging and in some cases via ultrasound [2,8]. In this report, we present a case of post-traumatic PCF formed following a motor vehicle crash and fully resolved after the placement of a tube thoracostomy. 

## Case presentation

A 33-year-old female with no known medical history presented to the emergency department via emergency medical services after a motor vehicle collision with a pole in which the patient was an unrestrained passenger. In the field, the patient was hypotensive to 70/43, tachycardic to 119 beats per minute, and agonally breathing. On arrival at the trauma bay, the patient’s Glasgow Coma Scale (GCS) was eight with two for eyes opening to painful stimuli, one for the absence of verbal response, and five for motor localization to painful stimuli. The patient was intubated in the trauma bay for airway protection and a cervical spine collar was applied. The secondary trauma survey demonstrated multiple lacerations to the right upper extremity, abrasions to the right posterior shoulder, right thigh, and right lateral chest wall, and ecchymosis overlying the right knee. 

**Figure 1:**
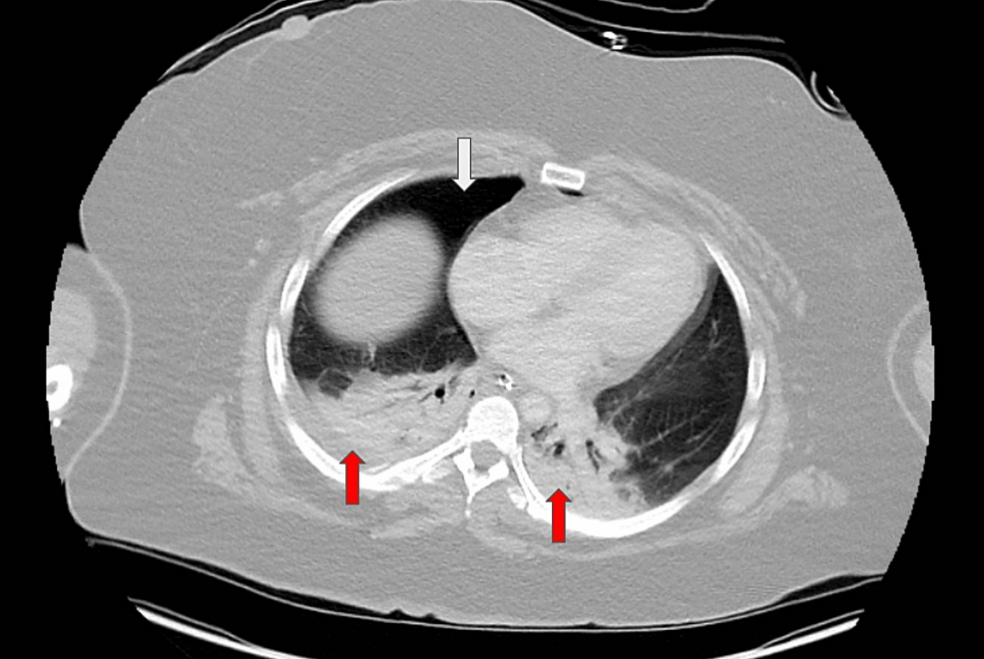
Axial CT with contrast shows right-sided pneumothorax (white arrow) and bilateral layering consolidations (red arrows).

**Figure 2:**
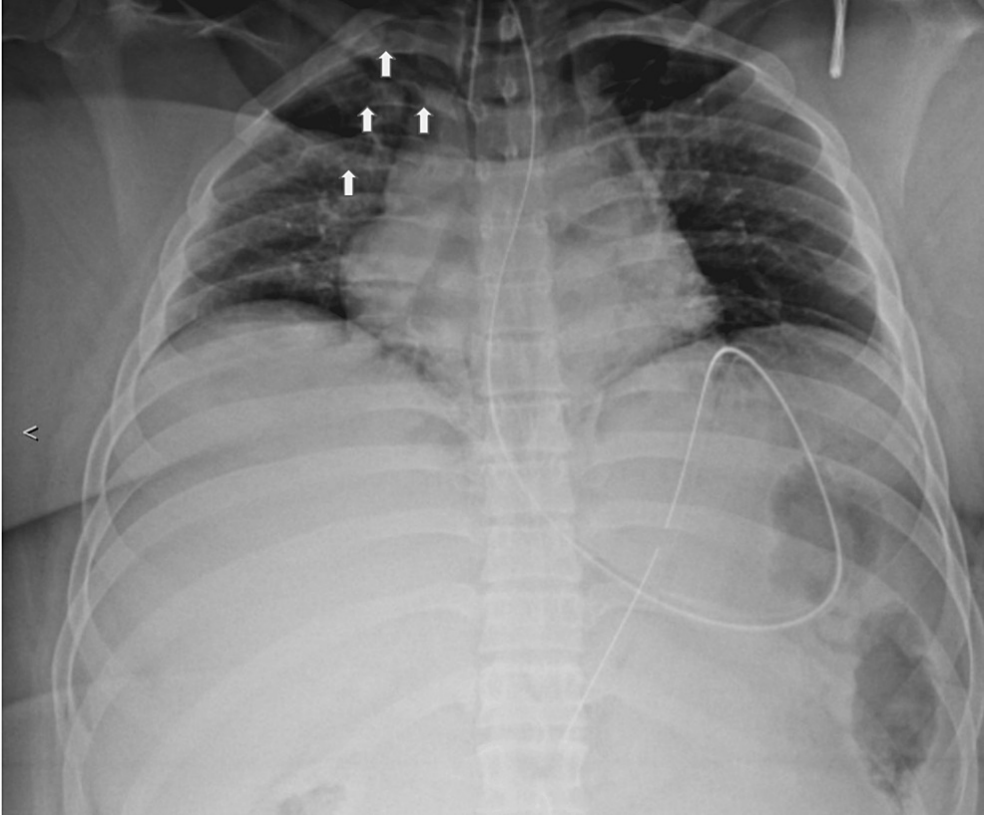
Portable chest X-ray shows fractures of the right second through fourth ribs (white arrows). Corresponding CT (Figure 1) confirmed fractures of the right first through seventh ribs, left first rib, and right L5 transverse process.

Initial focused assessment with sonography for trauma (FAST) exam was negative. Initial portable chest X-ray showed right-sided fractures of the second through fourth ribs, with bilateral consolidations. Non-contrast CT of the head and cervical spine were negative. The patient’s initial non-contrast CT of the chest abdomen and pelvis confirmed fractures of ribs one through seven on the right, and a fracture of the first rib on the right, with a small right-sided hemopneumothorax and bilateral layering opacities suggestive of pulmonary contusion (Figures 1, 2). Differential enhancement of the liver parenchyma in the setting of trauma suggested vascular injury. Perisplenic fluid raised concern for grade 2 splenic injury. Fat stranding surrounding the distal transverse colon was noted, likely due to focal mesenteric injury. The hemoperitoneum was present with fluid found in the pelvis, perisplenic region, hepatorenal space, and paracolic gutters. A non-displaced fracture of the right L5 transverse process was also found. CT angiogram of the neck was negative for traumatic vascular injury.

The patient was resuscitated with three total units of packed red blood cells, three units of fresh frozen plasma, and intravenous (IV) crystalloid fluids. Interventional radiology performed a proximal splenic artery embolization and hepatic angiography that was negative for active contrast extravasation. Additional traumatic injuries sustained included a class V trauma deceleration injury to the right kidney with complete devascularization and a right scapular fracture, both of which were determined to be non-operative.

**Figure 3:**
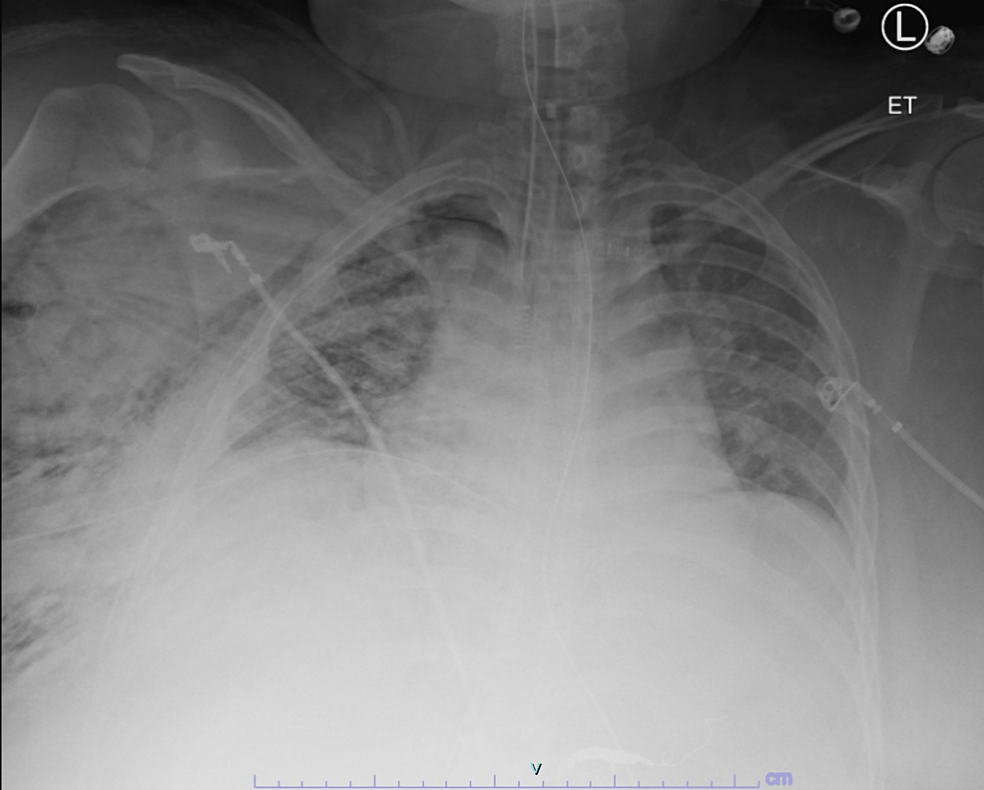
Portable chest X-ray taken immediately after tube thoracostomy shows the extent of the right-sided subcutaneous emphysema.

**Figure 4:**
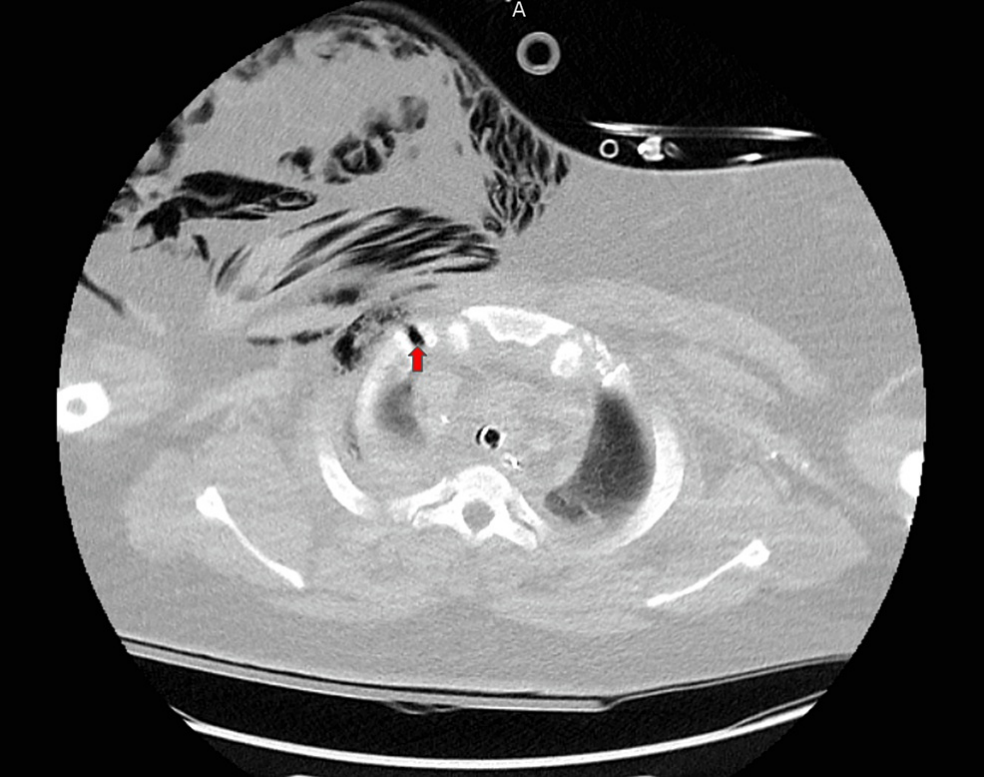
Axial CT with contrast shows the right-sided pleurocutaneous fistula (red arrow) with resulting subcutaneous emphysema of the right chest.

Follow-up chest X-ray (Figure 3) approximately 12 hours after hospital arrival showed subcutaneous emphysema of the right chest wall with the corresponding CT (Figure 4) showing worsening of the right-sided pneumothorax, as well as an intercostal PCF along the anterior superior chest wall.

**Figure 5:**
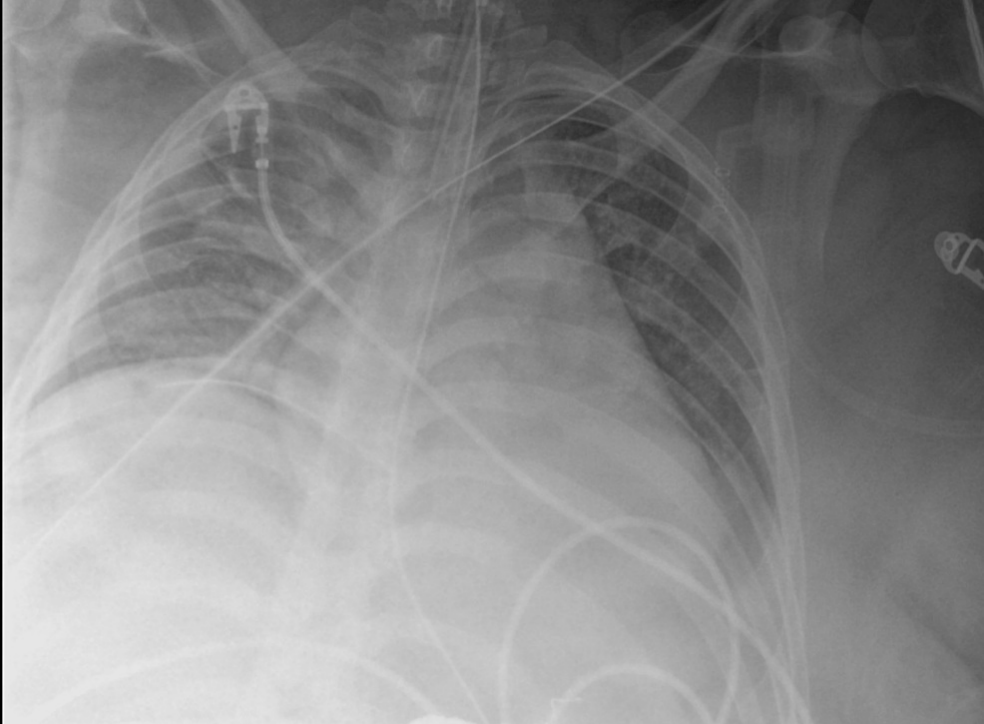
Portable chest X-ray 36 hours after tube thoracostomy placement shows near total resolution of the right-sided subcutaneous emphysema.

**Figure 6:**
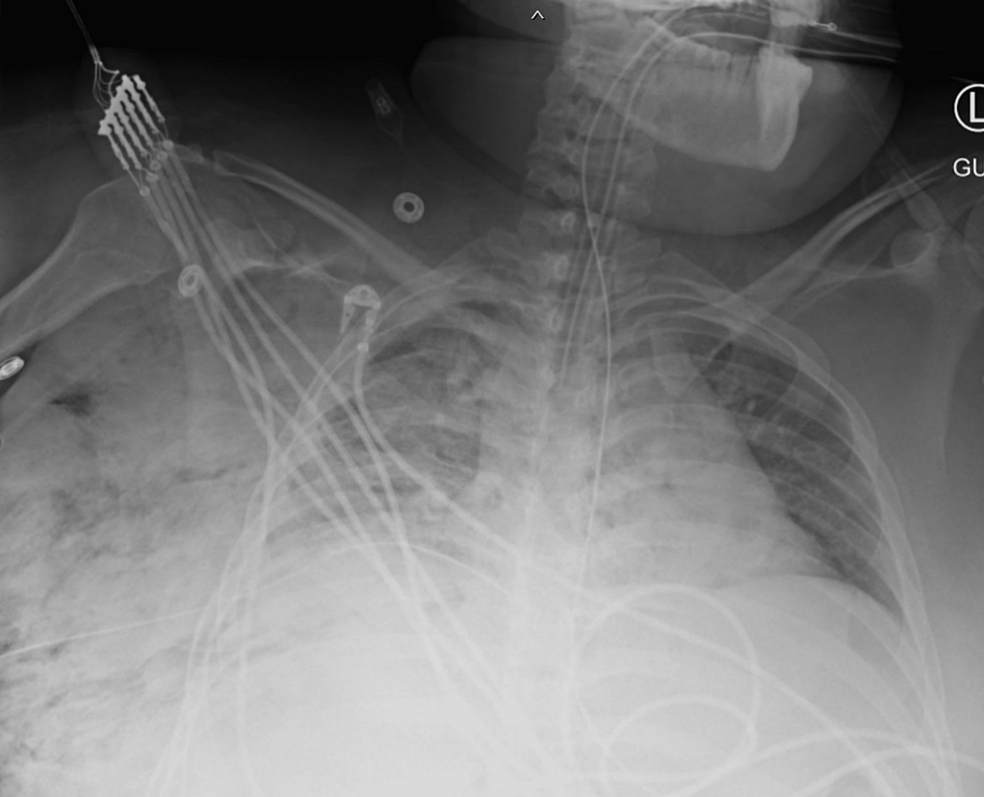
Portable chest X-ray taken 12 hours after tube thoracostomy shows improvement of right-sided subcutaneous emphysema.

A right-sided chest tube was placed and progression of the pneumothorax and subcutaneous emphysema was monitored, with improvement seen within one day (Figure 5) and near resolution occurring in less than 48 hours (Figure 6). Extubation was performed on hospital day 9 and the patient clinically improved with supportive care in addition to physical and occupational therapy. The patient was ultimately discharged to home on hospital day 16 with scheduled outpatient follow-up. 

## Discussion

Trauma is a major contributor to mortality throughout the world, with it being the leading cause of death under 50 years old in developed nations [1]. Up to 20% of these deaths are directly due to thoracic trauma, and up to 50% may be indirectly related to thoracic trauma [1]. It is important to promptly diagnose and treat thoracic trauma, as damage to key oxygen-circulating structures such as the heart, lungs, and great vessels may potentially lead to damage of extra-thoracic organs as well as death [1]. Due to this, trauma patients are assessed using the Advanced Trauma Life Support (ATLS) protocol. This protocol includes a primary, secondary, and tertiary survey. The primary survey is commonly known as the ABCDEs, which is the exam and associated interventions related to the patient’s airway, breathing, circulation, potential disability, and exposures of the patient’s body to evaluate for other immediate life-threatening injuries [10]. The secondary survey is an even more thorough head-to-toe examination, which includes the FAST exam as well as chest and pelvic X-rays [10]. At this time, further intervention and evaluation can occur. Within 24 hours of this process, the tertiary survey will occur, which is a repair of the primary and secondary surveys to reassess for anything that may have been missed [10]. This is a critically important aspect of the exam since many thoracic injuries may manifest over an extended period of time. This was seen in our patient, whose pneumothorax and PCF were found on repeat examination. 

Specific thoracic injuries that trauma units assess for can be divided into blunt and penetrating injuries, though both can be present at the same time [1]. Both types transmit an extraordinary amount of energy into the body, causing direct injury, as well as distal injury [1]. The most severe cardiac injuries include septal and free wall rupture, which are considered perimortal findings [1]. Other cardiac injuries range from mild contusions to arrhythmias, coronary artery injury, valvular defects, and wall motion abnormalities [1]. Aortic injury is also a common and feared finding in trauma patients. Tears and transections of the aorta occur due to the rapid deceleration experienced in the setting of trauma. These injuries are typically found in the aortic isthmus, slightly distal to the origin of the left subclavian artery, and have a mortality rate of nearly 100% if left untreated [1]. The lung parenchyma is also highly vulnerable to injury, though the most common injury is pulmonary contusion. Pulmonary contusion is seen in up to 70% of thoracic trauma patients but usually resolves within three to 10 days [1]. Chest wall injuries contribute significantly to parenchymal injuries of the lung seen in this patient population. Rib fracture is seen in close to half of these patients, but may go undetected on X-ray imaging [1]. The segmental fracture of three or more contiguous ribs, known as flail chest, is crucial to identify as the broken edges of the ribs may puncture the pleural sac and cause a pneumothorax. This complication was seen in our patient, who fractured seven contiguous ribs resulting in a hemopneumothorax. Not only can the fractured rib fractures puncture the lung, but they can also penetrate the subcutaneous tissue which abuts the pleura in normal circumstances, causing PCF.

PCF is an uncommon condition that is typically reported as a compilation of pleural tuberculosis, foreign bodies, cancer, radiation therapy post-mastectomy, or procedures such as tube thoracostomy and VATS [3]. This case is unique because the inciting factor was a high-speed trauma causing multiple rib fractures, which were responsible for creating a tract between the pleural space and subcutaneous tissue. The fractures of the right first and second ribs were likely the specific trauma that created the fistula based on its location (Figure 4). The fistula did not develop until several hours after the patient’s presentation, allowing us to document its formation over a series of X-ray and CT studies. The resolution of the fistula was also documented through a series of daily chest X-rays following tube thoracostomy. Although not performed in this case, previously reported cases of PCF have demonstrated the utility of point-of-care ultrasound in visualizing subcutaneous emphysema in addition to concurrent thoracic pathology such as pleural effusion [8]. Though tube thoracostomy was successful, it is difficult to determine if the fistula has healed or if the expansion of the pleura back to the chest wall has sealed it off, meaning that repeat pneumothorax could cause the fistula to reappear.

## Conclusions

To conclude, PCF is a rare occurrence that is usually a complication of another condition or procedure and has the potential for marked morbidity and mortality in affected individuals. To our knowledge, this is the first case of post-traumatic PCF documented in the literature. This is also a unique case that allows for the illustration of the real-time evolution and resolution of PCF through a series of radiographic images.

## References

[1] Jones H, Reynolds J (2021). Thoracic trauma and related topics. Grainger & Allison’s Diagnostic Radiology.

[2] Lin MT, Shih JY, Lee YC, Yang PC (2008). Pleurocutaneous fistula after tube thoracostomy: sonographic findings. J Clin Ultrasound.

[3] Duarte-Ribeiro F, Dias C, Mota M (2017). Bronchopleural and pleurocutaneous fistula in HIV patient with pulmonary tuberculosis. IDCases.

[4] Parrikar A, Tandon A, Durgeshwar G, Safwan A, Rathore Y (2023). Pleuro-cutaneous fistula: a rare complication of tuberculosis. QJM.

[5] Kotok D, Karpov O (2019). A window to the lung: lobectomy complicated by chronic pleurocutaneous fistula. Chest.

[6] Molnar TF (2017). Thoracic trauma: which chest tube when and where?. Thorac Surg Clin.

[7] Samuel LM, Kunkler IH, Dixon JM, Walker WS (1997). Pleurocutaneous fistula as a complication of radiation treatment in locally advanced breast cancer. J R Coll Surg Edinb.

[8] Amini R, Amini A, Hollinger P, Rhodes SM, Schmier C (2016). Emergency department diagnosis of a concealed pleurocutaneous fistula in a 78-year-old man using point-of-care ultrasound. World J Emerg Med.

[9] Krumpe P, Finley T, Wong L, Treasure R (1981). The bronchial leak squeak: a new sign for the physical diagnosis of bronchopleurocutaneous fistula. Chest.

[10] Morris SC (2009). The team approach to management of the polytrauma patient. Virtual Mentor.

